# Development of small‐scale models to understand the impact of continuous downstream bioprocessing on integrated virus filtration

**DOI:** 10.1002/btpr.2962

**Published:** 2020-02-03

**Authors:** Scott Lute, Julie Kozaili, Sarah Johnson, Kazuya Kobayashi, Daniel Strauss

**Affiliations:** ^1^ U.S. FDA CDER/OPQ/OBP/DBRR 2 Silver Spring Maryland; ^2^ Asahi Kasei Bioprocess America Glenview Illinois

**Keywords:** biotechnology, continuous manufacturing, small‐scale models, viral filtration, virus clearance

## Abstract

We designed small‐scale virus filtration models to investigate the impact of the extended process times and dynamic product streams present in continuous manufacturing. Our data show that the Planova 20N and BioEX virus filters are capable of effectively removing bacteriophage PP7 (>4 log) when run continuously for up to 4 days. Additionally, both Planova 20N and BioEX filters were able to successfully process a mock elution peak of increased protein, salt, and bacteriophage concentrations with only an increase in filtration pressure observed during the higher protein concentration peak. These experiments demonstrated that small‐scale viral clearance studies can be designed to model a continuous virus filtration step with specific process parameters.

## 1 INTRODUCTION

Biotechnology manufacturing has gradually evolved over the years from traditional batch mode operation to more continuous modes of operation with the implementation of continuous perfusion cell culture.^1^, ^2^ Recently, with technical advancements in continuous chromatography and an increased focus on single‐use systems, focus has shifted to an integrated upstream and downstream continuous process. However, due to technical constraints and lack of small‐scale models, most theoretical continuous manufacturing designs focus on a hybrid continuous system with one or more dedicated virus removal/inactivation steps remaining in batch mode via traditional hold tanks (e.g., low pH inactivation) or as a dedicated offline step (e.g., virus filtration). To facilitate the design and implementation of a fully continuous downstream process, one must understand the differences and challenges between traditional batch mode purification and integrated continuous purification. In particular, it is necessary to understand how these differences can impact the performance of each unit operation and how to design small‐scale studies of each continuous unit operation. Recent studies have focused mostly on implementation of continuous capture chromatography and continuous viral inactivation^3^, ^4^ with little research on the integration of virus filtration into continuous processes, aside from theoretical process design strategies.^5^

Virus filtration, also called nanofiltration, is an effective process step for removal of small parvovirus‐sized or larger virus particles, predominantly through a size‐based mechanism.^6^ Virus filters are single use and are typically run under constant pressure to a target throughput. This strategy has proven to be robust and effective despite the discovery of a few technical vulnerabilities and failure modes.^7^, ^8^ However, adapting a batch mode filtration strategy to continuous processes may prove challenging. Understanding how the unique technological parameters of continuous processing impact the performance of virus filters may lead to the development of both integration strategies and small‐scale process models.

To develop a small‐scale model for continuous virus filtration, it is necessary to consider two key differences between the batch unit operation and the integrated continuous unit operation. The first key difference is the concept of a discrete input and output versus dynamic input and output. In traditional batch mode purification, the downstream process flow path typically has hold tanks between each process step which allows for (a) load homogeneity, (b) discrete input volumes/concentrations for the subsequent unit operation, (c) control of optimized flow rate and pressure for each unit operation and for the virus filtrations step in particular, and (d) accommodation for filter replacement based on total throughput limits per filter (e.g., <1,000 L/m^2^) or processing time (e.g., <8 hr). For an integrated continuous purification process, there are no traditional hold tanks and the fluid flow is constant from one unit operation to another, creating a dynamic product stream with fluctuations in protein concentration, pH, and conductivity due to the inherent periodic elution peaks from one or more bind and elute steps. While these fluctuations may be dampened with the use of surge tanks, in instances where no surge tank is implemented, the fluctuating fluid streams have the potential to negatively impact virus filter performance. Previous studies have shown that some virus filters are susceptible to virus particle passage or reduced throughput with high protein concentrations or high ionic strength buffers.^9^, ^10^, ^11^ Additionally, in the unlikely event of a contamination, an elution peak may theoretically contain an increased concentration of virus, which may lead to a reduction in virus removal due to filter membrane overloading.^7^, ^12^ The second key difference is the system process parameters such as flow rate and pressure. In batch mode, each unit operation is a discrete process step that can be operated under optimal flow rates, process times, or pressure. In continuous mode, the process steps and flows are linked with the whole system flow typically governed by the flow rate of the continuous capture step. This difference can result in a virus filter operated for an extended processing time under low flow and/or low‐pressure parameters, as well as pressure fluctuations due to periodic elution peaks, which may be a concern for viral safety.^8^, ^13^, ^14^ Current small‐scale virus filtration models are not designed to accommodate dynamic loads or extended processing times due to system limitations or virus spike stability. In order to integrate a virus filter into a continuous process, new small‐scale designs may need to be developed to incorporate elements of continuous processing.

In these studies, we evaluated virus filter capabilities using a mammalian virus surrogate, bacteriophage PP7, by mimicking both the extended processing time and the fluctuating product load stream of a continuous virus filtration unit operation. To ensure representation of filters used in current manufacturing processes, we used a representative older model parvovirus filter (Planova 20N) and a newer model parvovirus filter (Planova BioEX) in our filter studies. Our goal was to determine the impact of a continuous process on the filtration operating parameters typically used in biotechnology processes and viral clearance capabilities of virus filters. The flow rates chosen for the studies presented in this work were dictated by the desire to maintain the pressures on the filters within the recommended limits established by the filters manufacturer when applicable (Planova BioEX) or from historical or published data (Planova 20N).^14^

## 2 MATERIALS AND METHODS

### 2.1 Filters

Planova 20N and BioEX filters (0.001 m^2^ effective surface area) from Asahi Kasei Medical (Tokyo, Japan) were used for the extended processing and peak mimicking studies. Nalgene Rapid‐Flow PES bottles (0.2 μm) from Thermo Fisher Scientific (Waltham, MA) were used as prefilters.

### 2.2 Extended processing studies

#### 2.2.1 Proteins, buffers, and reagents

Human immunoglobulin (h‐IgG; 50 mg/ml) was purchased from SeraCare Life Sciences (Milford, MA). Sodium acetate, sodium chloride, and sodium hydroxide were purchased from Fisher Scientific (Suwanee, GA).

#### 2.2.2 Viruses and assays

Crude bacteriophage PP7 was prepared and plaque assays were performed according to PDA TR‐41: Virus Filtration.^6^ Load material was freshly spiked with PP7 at a target 10^6^ PFU/ml at the start of each day, prefiltered using 0.2 μm Nalgene Rapid Flow PES bottles, and applied to the virus filters daily for 4 days. Aliquots of spiked feed and 12 ml samples of the filtrate collected daily were stored at −80°C until plaque assays were conducted. Proportions of the filtrates from each day were pooled together to create a simulated pool at the end of each filtration. Large volume testing was performed on simulated pool samples. Log_10_ reduction values (LRV) were calculated by subtracting the log_10_ of the total virus in the filtrate from the log_10_ of the virus load applied to the filter.

#### 2.2.3 Equipment and setup

**Figure 1:**
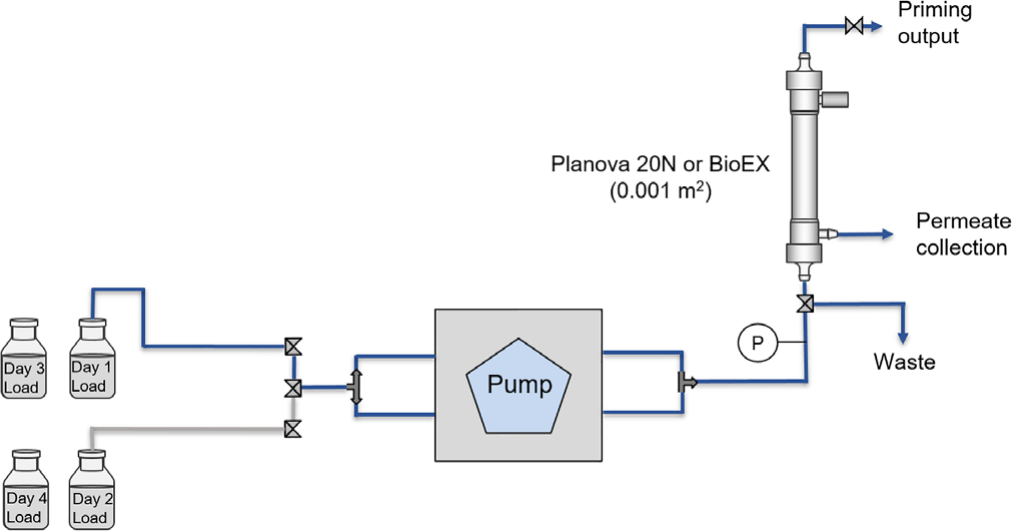
Schematic of virus filtration setup with two load reservoirs that are switched daily

In order to conduct continuous filtration experiments for up to 4 days, the filtration setup was configured to have two load reservoirs connected to the feed line with a three‐way valve that can be switched between the reservoirs daily to allow uninterrupted virus filtration while supplying freshly spiked load material daily (Figure 1). The flow, split between two lines, was controlled by a Masterflex peristaltic pump (Easy‐Load II) with two pump heads to reduce pressure fluctuations and PharMed BPT size 13 tubing (Masterflex, Vernon Hills, IL). Pressure monitored with a sensor placed at the lower inlet nozzle of the virus filter was recorded by a SciLog PureTec (Parker Hannifin, Cleveland, OH).

#### 2.2.4 Filtration conditions

Virus filtration runs were conducted at constant flow starting at around 7 and 28.4 psi for Planova 20N and BioEX filters, respectively. Filtrations were ended if the pressure reached the manufacturer recommended pressure limits of 14.2 or 49.7 psi, respectively. Prior to filtration, the filter was primed with 10–15 ml of deionized water. For each filter type, commercially available h‐IgG was diluted at a relatively low concentration due to low purity of the material in 50 mM acetate and 20 mM sodium chloride buffer at pH 6.0. The diluted h‐IgG solution was prepared for each filtration and stored at 4°C for the duration of the run. Aliquots were obtained daily, spiked with PP7, pre‐filtered, then loaded into the virus filter at a specific flow rate (Table 1). The load was switched to a freshly spiked container once daily to ensure consistent PP7 loading throughout the run. The filtrate was collected once daily as well. All runs were performed in duplicate.

**Table 1:** Extended processing filtration conditions for each filter type

Filter type	h‐IgG load (g/L)	Flow rate (ml/min)	Flux (LMH)
Planova 20N	0.150	0.5	30
Planova BioEX	0.025	1.2	72

### 2.3 Peak mimicking studies

#### 2.3.1 Proteins, buffers, and reagents

The peak mimicking studies had three components of the process fluid (protein concentration, salt concentration, and PP7 concentration) adjusted to create a buffer peak across the filters using two different load materials, A and B. The diluted h‐IgG concentrations and buffer concentrations can be seen in Table 2.

**Table 2:** Experimental conditions for peak mimicking studies

Experiment^a^	Load A	Load B
High protein	1 mg/ml h‐IgG 50 mM acetate, 20 mM sodium chloride pH 6.0 PP7, ~10^7^ PFU/ml	10 mg/ml h‐IgG 50 mM acetate, 20 mM sodium chloride pH 6.0 PP7, ~10^7^ PFU/ml
High salt		1 mg/ml h‐IgG 50 mM acetate, 500 mM sodium chloride pH 6.0 PP7, ~10^7^ PFU/ml
High phage	1 mg/ml h‐IgG 50 mM acetate, 20 mM sodium chloride pH 6.0 PP7, ~10^6^ PFU/ml	1 mg/ml h‐IgG 50 mM acetate, 20 mM sodium chloride pH 6.0 PP7, ~10^8^ PFU/ml
Triple spike		10 mg/ml h‐IgG 50 mM acetate, 500 mM sodium chloride pH 6.0 PP7, ~10^8^ PFU/ml

a Planova 20N filters run at 0.5 ml/min, Planova BioEX filters run at 1.0 ml/min.

#### 2.3.2 Viruses and assays

Load materials A and B were freshly spiked with PP7 to the target shown in Table 2 at the start of each day, prefiltered using 0.2 μm Nalgene Rapid‐Flow PES filter bottles and applied to the virus filters. The target spike for the high protein and high salt concentration runs was 10^7^ PFU/ml for both load materials A and B. For high phage and the triple spike runs, load material A had a PP7 spike target of 10^6^ PFU/ml, while load material B had a PP7 spike target of 10^8^ PFU/ml to provide a 100‐fold increase in virus particles without overloading the filter membrane.^7^ Spiked load materials were sampled and held at room temperature while filtrate samples were collected. Both load and filtrate samples were assayed for titer using PP7 plaque assay immediately after each run. Remaining sample volumes were subsequently stored at 4°C for repeated plaque assays, if required.

#### 2.3.3 Equipment and setup

**Figure 2:**
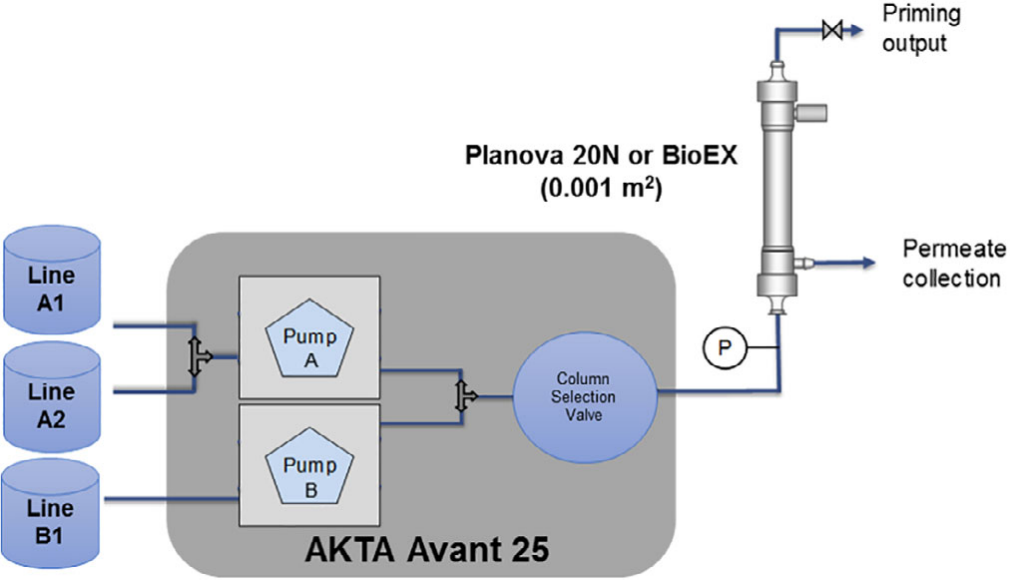
Schematic of virus filtration setup for peak mimicking studies

**Figure 3:**
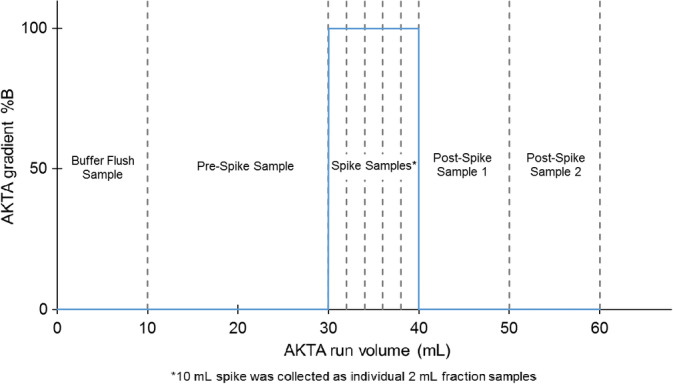
Manual sampling schedule for both Planova 20N and BioEX runs

In order to mimic the properties of an elution peak, the virus filters were connected to the column selection valve of an AKTA Avant 25 (GE Healthcare Life Sciences), a baseline load was applied using pump A, and a step gradient of the simulated elution peak was applied using buffer pump B (Figure 2). Samples were manually collected, as shown in Figure 3, for each filtration experiment. The AKTA Avant was programmed with the Unicorn 6.1 software to run the following sequence for each filter experiment:

- 10 ml of inlet line A1 (Flush Buffer: 50 mM acetate, 20 mM NaCl pH 6.0)
- 20 ml of inlet line A2 (Load A)
- 10 ml Step Gradient: 100% line B1 (Load B)
- 20 ml of inlet line A2 (Load A)

Similar to the extended processing studies, filtration runs using Planova 20N and BioEX filters (0.001 m^2^) were performed at constant flow rate of 0.5 and 1.0 ml/min (30 and 60 LMH), respectively. Flow rates were set to avoid exceeding manufacturer recommended pressure limits. Pressure was monitored by the AKTA “Pre‐Column” pressure sensor and/or using in‐line pressure sensors with external pressure monitor (SciLog PureTec) connected between the column selection valve and the filter. Each filter was primed with at least 20 ml of deionized water, all AKTA inlet lines and flow paths were sanitized with 0.5 M NaOH, and all buffer inlet lines were primed with respective buffers (Table 2) prior to starting the filtration program. Samples were manually collected into sterile 15‐ml Falcon tubes for spike samples or 50‐ml Falcon tubes for all other samples. The AKTA fraction collector was not used to minimize potential contamination and to minimize backpressure from AKTA tubing. All experiments were performed in duplicate, with select conditions having a third run with an in‐line pressure sensor to verify the pressure readings from the AKTA and to determine the impact of the in‐line pressure sensor on filtration. Additionally, each experiment had one representative run without sampling whereby the virus filter was integrated into the AKTA Avant system with the permeate flow connected to the AKTA UV and conductivity sensors to allow collection of data and assure that a mimicked elution peak was achieved.

## 3 RESULTS AND DISCUSSION

### 3.1 Extended processing studies with Planova 20N filters

**Figure 4:**
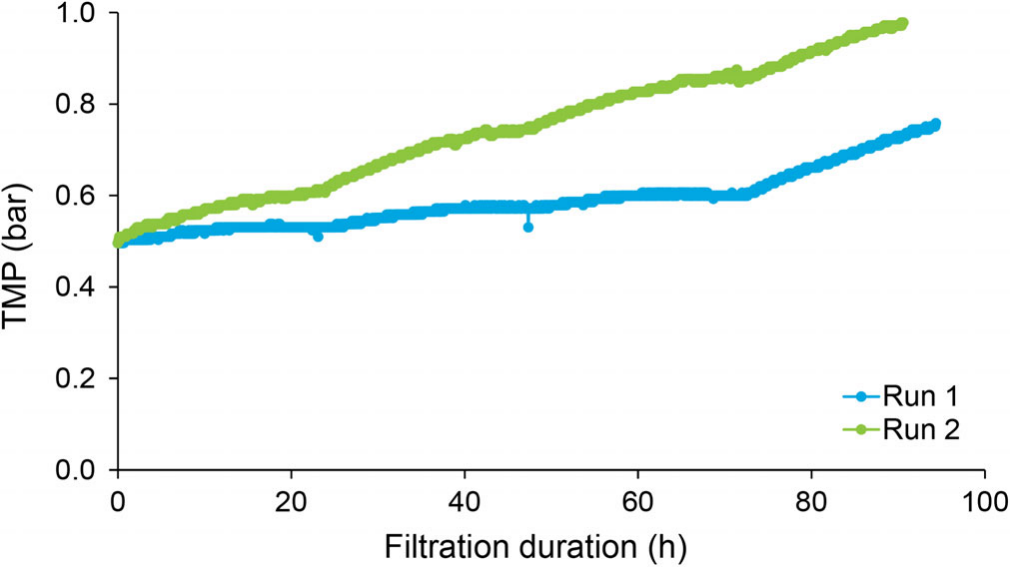
Pressure profile for Planova 20N filters during extended processing filtrations

The goal of these studies was to use Planova 20N filters in a continuous virus filtration setup. Planova 20N filtrations with PP7‐spiked h‐IgG solutions were carried out for 4 days. A starting pressure of 7 psi was chosen due to previously determined low‐pressure limit.^14^ The pressure at the end of the run reached 11 psi and 14.2 psi, for Run 1 and Run 2, respectively (Figure 4). The maximum recommended pressure for Planova 20N filters (14.2 psi) was not exceeded for either run. Since the use of pumps is known to create pressure fluctuations, we used two pump heads in this setup, which eliminated pressure fluctuations (data not shown). While the use of two pump heads for this small‐scale setup allowed for little to no pressure fluctuations, other measures may be deemed suitable for a large‐scale setup; a combination of appropriate tubing size and pump type is often available for manufacturing scale. The flux for both runs was around 30 L per meter squared per hour (LMH), as can be expected from a constant flow mode. The target PP7 spike for the runs was 10^6^ PFU/ml. For Planova 20N filtrations, a total of 2.9 L of material was filtered on each 0.001 m^2^ filter over 4 days, resulting in a total virus load of 9.5 log PFU (12.5 log PFU/m^2^). Load samples from each day (Days 0–3), filtrate samples from each day (Filtrates 1–4), and a simulated filtrate pool sample were collected. PP7 titers were obtained using plaque assay. Large volume testing was performed on the filtrate pool samples. Titer results and calculated PP7 LRV are presented in Table 3. The daily load of freshly spiked material ensured a consistent virus load of 5.7–6.0 log PFU/ml, as PP7 stability was found to decrease after 24 hr under the experimental solution conditions used (data not shown). This fresh daily spike may not be necessary under different solution conditions or if a different model virus were to be used. All filtrate samples showed complete clearance with a calculated PP7 LRV of ≥6.0 log PFU/ml for Run 1 and ≥6.1 log PFU/ml for Run 2. The results show that Planova 20N filters can be used in a continuous processing setup and achieve effective virus removal. The overall throughput for each of these runs was 2,900 L/m^2^, well above what can be expected from a batch process.

**Table 3:** PP7 titer and PP7 LRV for Planova 20N (P20N) and BioEX filters for extended processing studies

Sample	Log titer (PFU/ml)			
	P20N—Run 1	P20N—Run 2	BioEX—Run 1	BioEX—Run 2
Load range	5.7–5.9	5.8–5.9	5.9–6.0	4.2–4.3
Day 1 filtrate	≤0.78	≤0.78	≤0.78	≤0.78
Day 2 filtrate	≤0.78	≤0.78	≤0.78	≤0.78
Day 3 filtrate	≤0.78	≤0.78	≤0.78	≤0.78
Day 4 filtrate	≤0.78	≤0.78	≤0.78	≤0.78
Filtrate pool	≤−0.22	≤−0.22	≤−0.22	≤−0.22
Pool LRV	≥6.0	≥6.1	≥6.1	≥4.5

While both Planova 20N filtrations were completed within the recommended pressure limits, a difference in the pressure profile of the two runs is observed, specifically after the first day of filtration. This indicates a permeability decay as filtration time increases beyond 8 hr. These results demonstrate the complexity of running a virus filter in a continuous setup as well as the need for better controls for the process. Additional controls may include installation of a bubble trap in‐line before the filters in order to prevent any microscopic bubbles in the load solution from affecting filter performance. Temperature control is also one of the parameters to consider for future studies. Finally, prefilter optimization or the inclusion of the prefilter inline could improve fouling contaminants.

### 3.2 Extended processing studies with Planova BioEX filters

**Figure 5:**
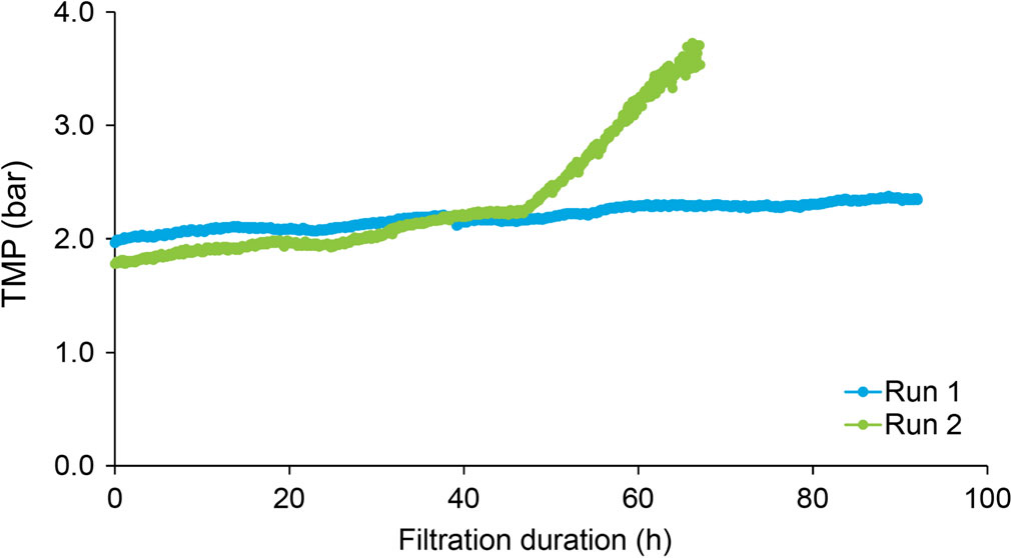
Pressure profile for Planova BioEX filters during extended filtrations

Planova BioEX filtrations were performed using the conditions previously described and shown in Table 1. For Planova BioEX filters, a manufacturer minimum recommended pressure of 28.4 psi had to be achieved at the start of each filtration, thereby dictating the flow rate for the runs. For Run 1, a flow rate of 1.2 ml/min was found to achieve a starting pressure of 28.4 psi. Run 1 was conducted for 4 days and ended with a pressure of 34 psi. For Run 2, a decision was made to use the same flow rate as Run 1in order to minimize flow variations between the runs. The starting pressure was 26 psi. After load replacement on the third day, the pressure quickly increased to reach the maximum recommended pressure for Planova BioEX filters (49.7 psi). The run was stopped after 3 days of filtration. The pressure profile is presented in Figure 5. Both runs showed a stable flux of 72 LMH.

For Planova BioEX Run 1, a total of 6.9 L of material was loaded on the 0.001 m^2^ filter over 4 days, resulting in a total virus load of 9.8 log PFU (12.8 log PFU/m^2^). For Run 2, a total of 5.2 L of material was filtered over 3 days, resulting in a total virus load of 8.0 log PFU (10.5 log PFU/m^2^). Samples were collected as described for Planova 20N filtrations. Large volume testing was performed on the filtrate pool samples. Titer results and calculated PP7 LRV are presented in Table 3. All filtrate samples showed complete clearance with a calculated LRV of ≥6.1 log PFU/ml for Run 1 and ≥4.5 for Run 2. The lower PP7 LRV in Run 2 is related to the lower load range for the run.

In a traditional batch mode virus filtration step, the expectation is that two runs with similar load materials would result in similar pressure profiles. The same cannot be said of long‐duration virus filtrations. The performance of the Planova BioEX filters over two runs appears identical over 2 days (48 hr of filtration), but in this case a small load variation resulted in a greater difference in the pressure profile. Future control measures were discussed in the previous section for Planova 20N filters and can also be implemented for Planova BioEX filtrations in the future. Overall, the results show that Planova BioEX filters can be used in a continuous processing setup and achieve effective virus removal. The observed throughout from these runs was between 5,200 and 6,900 L/m^2^, still achieving a greater throughput than a traditional batch process.

### 3.3 Peak mimicking filtrations for Planova 20N and BioEX filters

**Figure 6:**
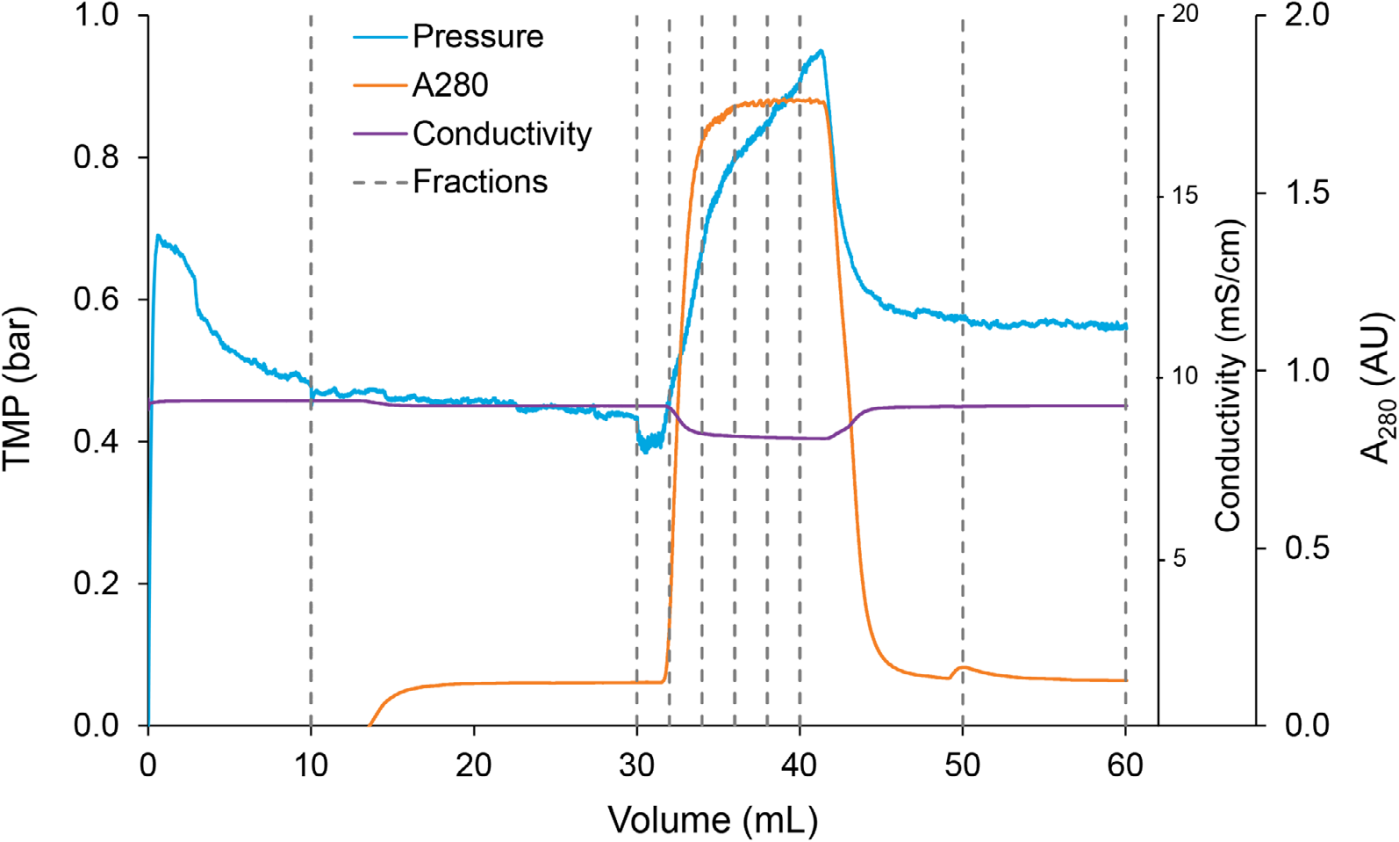
Representative elution profile for peak mimicking filtration using a Planova 20N filter

**Figure 7:**
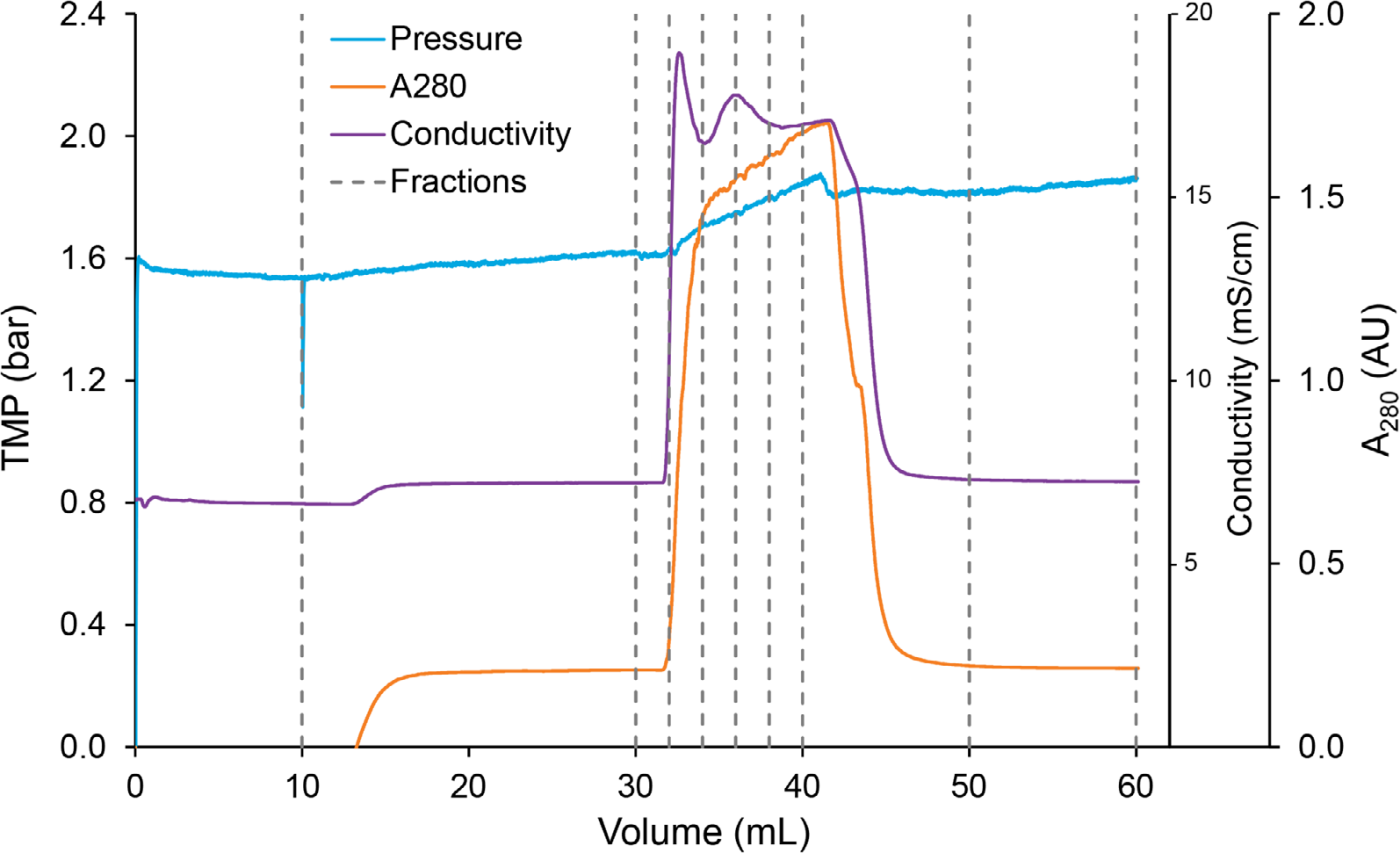
Elution profile for peak mimicking filtration using a Planova BioEX filter

In a continuous and integrated downstream platform, there is a potential to have periodic fluctuations of the fluid stream eluting from the previous chromatography steps, particularly if a buffer exchange or a peak dampening step is not included prior to virus filtration. While there is extensive knowledge and research on virus filtration, these data are based on well‐defined homogenous feed streams, not a heterogenous and fluctuating feed stream. The goal of these studies was to understand the impact that a periodic elution peak, thus a heterogenous feed stream, may have on the virus filtration. Three factors that were determined to potentially spike in concentration in an elution peak and impact filter performance were protein concentration, conductivity and virus titer. As shown in Table 2, these factors were tested as single independent variables and also combined as one total peak. The concentration levels for Load B were chosen as worst‐case conditions. For all experiments, the total volumetric throughput was 50 L/m^2^ with the first 20 L/m^2^ consisting of a baseline load of PP7‐spiked h‐IgG followed by 10 L/m^2^ mock peak and finally 20 L/m^2^ of baseline load, as mentioned previously. Because of the potential for the Load B spike to cause a pressure increase, experimental flow rates were selected that allowed for a significant increase in pressure without exceeding the filter limit. For the Planova 20N experiments, a flow rate of 0.5 ml/min (flux of 30 LMH) provided a baseline pressure of approximately 7 psi. For Planova BioEX experiments, a flow rate of 1.0 ml/min (flux of 60 LMH) provided a baseline pressure of approximately 20 psi. For both Planova 20N and BioEX filters, the Avant pump programming was able to successfully recreate elution peaks as seen by the conductivity and UV traces obtained when the filters were connected so the permeate flowed through the Avant sensors (Figures 6 and 7). However, there was a noticeable 2–4 ml delay between the initiation of Load B and the presence of Load B in the filtrate, which can be attributed to the AKTA mixer, flow path, and filter hold up volume. Additionally, the inclusion of a SciLog in‐line pressure sensor demonstrated that the AKTA pressure sensor is sufficiently accurate to monitor transmembrane pressure, and results in a slightly higher pressure measurement at the faster flow rate (Figure 7). Peaks in buffer conductivity and PP7 titer alone had minimal impact on filtration pressure (data not shown); while the runs spiked with high protein concentration, including the triple spike, had a noticeable pressure increase. Pressure increased to 14 psi with the Planova 20N filters and 26.5 psi with the Planova BioEX filters. The pressure increase was mostly reversible with a reduction in pressure after the load was switched back to Load A; however, in some instances, pressure did not return to baseline (Figure 6). While the increase in baseline pressure had no impact for a single mock peak, sequential peaks being filtered across both the Planova 20N and BioEX filters may cause the pressure limit to be reached prematurely. This risk can be mitigated by oversizing the filter, by peak attenuation from an in‐line mixer, single‐pass tangential flow filtration unit, or surge vessel, for example. Despite the concerns of a rising baseline pressure, both filters demonstrated the ability to process a dynamic fluid stream that may be present in a fully continuous and integrated downstream purification platform. It should also be noted that the increase in pressure did not go above recommended operating pressures for the filters.

In addition to monitoring pressure for filter performance evaluation, filtrate samples were manually collected throughout each experiment as shown in Figure 3 and assayed for PP7 titer by plaque assay. As virus carryover is a concern in AKTA systems, the buffer flush sample served as a negative control and an installation check. Any filtration run with PP7 detected in the buffer flush sample was discarded, the system was re‐sanitized, and the run was repeated with a new filter and new buffers. The pre‐spike sample of 20 ml provided a baseline load of protein and PP7 to the filter prior to the spike challenge. The 10 ml of spike load was fractioned into 5 × 2 ml samples to track the potential PP7 passage as the mock peak passed through the filter. The final 20 ml of baseline load were collected in 2 × 10 ml fractions, Post‐Spike 1 and Post‐Spike 2 (Figures 6 and 7). It should be noted that Post Spike 1 samples were designed to collect residual spike load and contain a ratio of the Load A and Load B fluid stream due to inherent hold‐up volume of the AKTA system and the filter. Post Spike 2 samples contain only Load A.

Planova 20N filter studies were run at a constant flow rate of 0.5 ml/min for a total run time of 120 min post flushing. For the Planova 20N filters, PP7 was detected in at least one duplicate run for each test condition with sample PP7 LRV ranging from 3.1 to >7.61 (Table 4). PP7 passage did not correlate with the increase in filtration pressure observed during the high protein and triple spike runs, as a few of the runs had no detectable plaques in the samples. However, there is a correlation between the total PP7 PFU loaded onto the filter and PP7 passage. As the total PP7 PFU loaded per filter increased to above approximately 10^9^ total PFU, an increasing number of plaques were detected in assays of the samples, regardless of test conditions (Table 4). These findings are consistent with the concept of designing viral clearance studies based on total virus challenge ^12^. While the Interestingly, in the case of triple spike‐Run 1 and Run 2, there were no plaques detected in the assays of Post‐Spike 2 pool samples despite detection of plaques in the assays of previous samples. This was not observed in the overloaded single spike high phage or high protein runs whereby PP7 were present in these samples at equivalent or higher concentrations. This suggests that the substantial retention of PP7 after the spike, when overloaded, may be due to a compound effect of the change in ionic strength and protein concentration. Further studies are required to fully understand this interaction. Overall, while PP7 passage post‐spike was observed, the data demonstrate that the older Planova 20N filters may be used in an integrated continuous downstream process with a dynamic fluid load. Effective viral clearance was achieved under recommended PP7 loads (<9 log PFU per filter), suggesting that proper design of small‐scale studies is required based on the virus being tested as parvoviruses, PPV and MMV, are smaller than PP7 and may report passage at a lower load threshold.

**Table 4:** PP7 load and removal by run on Planova 20N filters

Sample		High salt		High phage		High protein		Triple spike	
		Run 1	Run 2	Run 1	Run 2	Run 1	Run 2	Run 1^a^	Run 2^a^
Titer (log PFU/ml)	Load A	7.44	7.08	6.68	6.91	7.61	8.23	7.49	7.49
	Load B	8.06	7.22	8.84	8.18	7.68	8.03	9.37	9.37
LRV	Pre‐spike	>7.44	>7.08	>6.68	>6.91	>7.61	6.71	>7.49	>7.49
	Spike 1^b^	>7.06	>6.22	>7.84	>7.18	>6.68	5.59	6.98	>8.37
	Spike 2^b^	>7.06	>6.22	>7.84	>7.18	>6.68	5.54	6.66	8.37
	Spike 3^b^	>7.06	>6.22	>7.84	>7.18	>6.68	4.67	4.73	5.62
	Spike 4^b^	>7.06	>6.22	>7.84	>7.18	>6.68	5.82	4.24	4.33
	Spike 5^b^	>7.06	>6.22	>7.84	>7.18	>6.68	6.08	4.08	3.95
	Post spike 1	>7.44	>7.08	5.40	>6.91	>7.61	5.67	3.10	6.97
	Post spike 2	7.44	>7.08	4.88	>6.91	>7.61	4.93	>7.49	>7.49
Total phage (log PFU)		9.35	8.81	9.85	9.26	9.32	9.89	10.39	10.39

a Runs performed on same day with same load.

b Maximum sample test volume of 100 μl.

Planova BioEX filter studies were run at a constant flow rate of 1.0 ml/min to provide sufficient pressure, resulting in a total run time of 60 min post flushing. Due to the short run times, a single load was made for each study condition with distinct filter runs performed on the same day in series. The data from the Planova BioEX filters demonstrated robustness with respect to virus removal capabilities. There was no PP7 detected in any sample for any of the test conditions, with PP7 LRV ranging from >5.30 to >8.14 for peak fraction samples. This includes high protein and triple spike runs where the filter pressure increased during the mock spike and conditions where the total PP7 PFU loaded exceeded 10 log PFU (Table 5). These data suggest that Planova BioEX filters can be used in an integrated continuous process with dynamic fluid properties and achieve effective virus removal.

**Table 5:** PP7 load and removal by run on Planova BioEX filters

Sample		High salt^a^		High phage^a^		High protein		Triple spike^a^	
		Run 1	Run 2	Run 1	Run 2	Run 1	Run 2	Run 1	Run 2
Titer (log PFU/ml)	Load A	7.54	7.54	7.34	7.34	7.08	7.08	6.96	6.96
	Load B	7.57	7.57	9.14	9.14	6.30	6.30	7.85	7.85
LRV	Pre‐spike	7.24	>7.54	>7.34	>7.34	>7.08	>7.08	>6.96	>6.96
	Spike 1^b^	>6.57	>6.57	>8.14	>8.14	>5.30	>5.30	>6.85	>6.85
	Spike 2^b^	>6.57	>6.57	>8.14	>8.14	>5.30	>5.30	>6.85	>6.85
	Spike 3^b^	>6.57	>6.57	>8.14	>8.14	>5.30	>5.30	>6.85	>6.85
	Spike 4^b^	>6.57	>6.57	>8.14	>8.14	>5.30	>5.30	>6.85	>6.85
	Spike 5^b^	>6.57	>6.57	>8.14	>8.14	>5.30	>5.30	>6.85	>6.85
	Post spike 1	>7.54	>7.54	>7.34	>7.34	>7.08	>7.08	>6.96	>6.96
	Post spike 2	>7.54	>7.54	>7.34	>7.34	>7.08	>7.08	>6.96	>6.96
Total phage log PFU		9.24		10.16		8.70		9.03	

a Runs were performed on same day with same load.

b Maximum sample test volume of 100 μl.

## 4 CONCLUSIONS

For a truly continuous manufacturing process, all upstream and downstream manufacturing steps are integrated with a continuous flow of product, which is in contrast to numerous hold tanks in traditional batch mode. For process steps that remain similar to traditional batch manufacturing, such as virus filtration, the impact of both a dynamic fluid stream and extended processing times must be understood. While there has been recent published research on continuous chromatography and continuous viral inactivation, there has been little published data on the impact of continuous manufacturing on virus filtration. This study provides both novel ideas when considering small‐scale design for mimicking continuous virus filtration conditions, including extended filtration process times and dynamic loading conditions, and experimental data to support the implementation into a continuous manufacturing setting. The extended processing small‐scale investigational studies overcame the concern of virus spike stability by allowing for a daily refresh of virus spike with minimal impact on the filtration process. The small‐scale investigational dynamic load studies using an AKTA Avant system successfully mimicked an elution peak spike being processed through a virus filter. The data show that both Planova 20N and Planova BioEX filtration pressure is only impacted by high protein concentrations present in an elution peak. While the pressure is reduced after passage of the elution peak, baseline pressure is not always achieved post spike. Further experiments mimicking multiple elution peaks in series would be needed to test if pressure limits would be exceeded, limiting the lifetime of the virus filters, and to see if periodic pressure fluctuations can impact virus passage. In terms of virus removal capabilities, the results of the small‐scale studies demonstrate that the Planova 20N and BioEX filters are both robust with respect to extended processing times and mock elution peaks. The only factor of the mock elution peak affecting virus removal was total virus loaded onto the Planova 20N filter, a known concern^7^, ^12^ that can be mitigated by controlling the virus spike levels. While the data in this study support the use of Planova 20N and BioEX filters in a continuous purification system, it should be noted that the results are protein‐, buffer‐, and filter‐specific and were based on a worst‐case framework using a bacteriophage model virus. However, the small‐scale investigational models presented here can be utilized as a starting point to understand the impact of continuous processing on other virus filters and for other protein products. Further small‐scale validation models mimicking actual process conditions, and including model parvoviruses, still need to be developed. Lower spike levels may need to be considered when using such model parvoviruses. The goal from such studies would be to find a balance between the spike level as to not overload the virus filter and the viral clearance to be achieved. What these studies demonstrate is that viral clearance is robust and achievable despite the effects of subtle differences in feedstock on virus filter capacity. These effects will need to be further understood especially in light of the potential for perfusion processes going over multiple weeks.

## DISCLAIMERS

1. This manuscript reflects the views of the authors and should not be construed to represent FDA's views or policies.
2. This manuscript is not an FDA endorsement of one specific virus filter.
